# Toxicity of Heavy Metals and Recent Advances in Their Removal: A Review

**DOI:** 10.3390/toxics11070580

**Published:** 2023-07-03

**Authors:** Manar K. Abd Elnabi, Nehal E. Elkaliny, Maha M. Elyazied, Shimaa H. Azab, Shawky A. Elkhalifa, Sohaila Elmasry, Moustafa S. Mouhamed, Ebrahim M. Shalamesh, Naira A. Alhorieny, Abeer E. Abd Elaty, Ibrahim M. Elgendy, Alaa E. Etman, Kholod E. Saad, Konstantina Tsigkou, Sameh S. Ali, Michael Kornaros, Yehia A.-G. Mahmoud

**Affiliations:** 1 Botany Department, Faculty of Science, Tanta University, Tanta 31527, Egypt; manar.abdelnabi@ejust.edu.eg (M.K.A.E.); nehal31012429@science.tanta.edu.eg (N.E.E.); maha31012879@science.tanta.edu.eg (M.M.E.); shaimaa31012609@science.tanta.edu.eg (S.H.A.); shawqe31012093@science.tanta.edu.eg (S.A.E.); mostafa30974505@science.tanta.edu.eg (M.S.M.); ibrahem231058915@science.tanta.edu.eg (E.M.S.); nira231058550@science.tanta.edu.eg (N.A.A.); abeer31012615@science.tanta.edu.eg (A.E.A.E.); ibrahim31012107@science.tanta.edu.eg (I.M.E.); alaa231058671@science.tanta.edu.eg (A.E.E.); khaloud31012947@science.tanta.edu.eg (K.E.S.); yehiamahmoud@science.tanta.edu.eg (Y.A.-G.M.); 2 Biotechnology Program, Institute of Basic and Applied Science (BAS), Egypt-Japan University of Science and Technology, New Borg El-Arab City 21934, Egypt; 3 Microbiology Department, Faculty of science, Damanhour University, Behaira 22514, Egypt; s.abdelazeez00804@sci.dmu.edu.eg; 4 Department of Chemical Engineering, University of Patras, 1 Karatheodori str, 26504 Patras, Greece; ktsigkou@chemeng.upatras.gr; 5 Biofuels Institute, School of the Environment and Safety Engineering, Jiangsu University, Zhenjiang 212013, China

**Keywords:** heavy metals, bioremediation, heavy metals toxicity, biosorption, biomineralization, biotransformation

## Abstract

Natural and anthropogenic sources of metals in the ecosystem are perpetually increasing; consequently, heavy metal (HM) accumulation has become a major environmental concern. Human exposure to HMs has increased dramatically due to the industrial activities of the 20th century. Mercury, arsenic lead, chrome, and cadmium have been the most prevalent HMs that have caused human toxicity. Poisonings can be acute or chronic following exposure via water, air, or food. The bioaccumulation of these HMs results in a variety of toxic effects on various tissues and organs. Comparing the mechanisms of action reveals that these metals induce toxicity via similar pathways, including the production of reactive oxygen species, the inactivation of enzymes, and oxidative stress. The conventional techniques employed for the elimination of HMs are deemed inadequate when the HM concentration is less than 100 mg/L. In addition, these methods exhibit certain limitations, including the production of secondary pollutants, a high demand for energy and chemicals, and reduced cost-effectiveness. As a result, the employment of microbial bioremediation for the purpose of HM detoxification has emerged as a viable solution, given that microorganisms, including fungi and bacteria, exhibit superior biosorption and bio-accumulation capabilities. This review deals with HM uptake and toxicity mechanisms associated with HMs, and will increase our knowledge on their toxic effects on the body organs, leading to better management of metal poisoning. This review aims to enhance comprehension and offer sources for the judicious selection of microbial remediation technology for the detoxification of HMs. Microbial-based solutions that are sustainable could potentially offer crucial and cost-effective methods for reducing the toxicity of HMs.

## 1. Introduction

**Figure 1:**
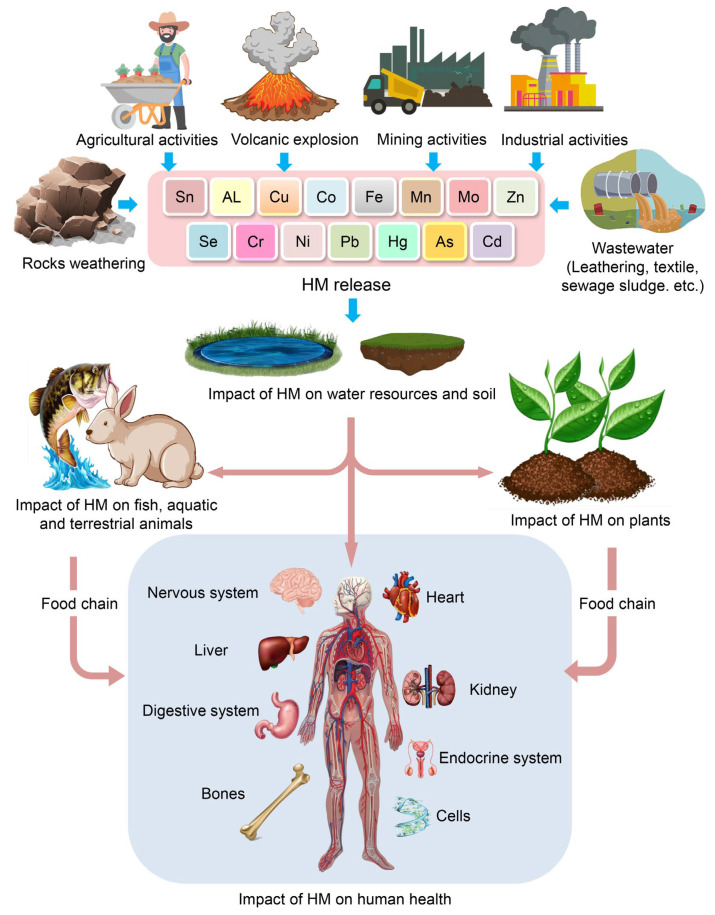
Origins and impacts of HMs on humans through the food chain.

Heavy metals (HMs) are defined as metallic elements with a greater density than water [1]. HMs are naturally occurring elements with a large atomic weight and at least five times the density of water [2]. Assuming that heaviness and toxicity are intertwined, HMs also include metalloids, such as arsenic (As), that can induce toxicity at low exposure levels [3]. Various heavy metals, such as chromium (Cr), cadmium (Cd), nickel (Ni), copper (Cu), zinc (Zn), lead (Pb), mercury (Hg), and As, are recognized as biologically dispensable and detrimental to the aquatic ecosystem [4]. Anthropogenic sources, including but not limited to the nonferrous metallurgical industry, mining, mineral processing, electroplating, leather tanning, and chemical industries, are commonly attributed to the discharge of hazardous metals such as HMs [5]. The level of toxicity exhibited by these hazardous materials is contingent upon the amount and length of time that living organisms are exposed to them. Figure 1 depicts the origins and impacts of HMs on humans through the food chain. Prolonged exposure to HMs through dermal contact, ingestion of contaminated food, and inhalation can result in the development of diverse disorders in both humans and other organisms [6]. In addition, the issue of soils contaminated with HM has become an increasingly significant environmental concern worldwide [7].

The presence of HMs in composting materials poses a considerable obstacle to their utilization. Consequently, there has been a significant focus on mitigating the toxicity of HMs during the composting process. The composting of HMs primarily impacts microorganisms, and the toxicity levels of different HMs exhibit variability [8]. Various techniques, including chemical, physical, or a combination of both, can be employed to degrade HMs. A considerable number of these techniques are neither ecologically sustainable nor financially viable. Microbial remediation is a viable alternative technique due to the efficient adaptability of microorganisms to their environment [9,10,11,12,13,14]. The remediation of HMs through microbial bioremediation involves various mechanisms, including bioaugmentation, biosorption, biomineralization, and biotransformation [15]. The implementation of various techniques such as remediation, microbial fuel cells, biofilm, nanomaterials, cell immobilization, and genetic engineering have shown great potential in the removal of HMs. The aim of this review is to provide updated knowledge regarding the abundance of HMs and their potential toxicity on living organisms and plants, as well as their negative impact on human health. It additionally offers mechanistic insights and highlights research uncertainties pertaining to HM remediation via biological and green remediation approaches.

## 2. Impact of HMs on Human Health

The exposure of humans to HMs is a result of industrial activities and can manifest through various means, such as ingestion, inhalation, and dermal absorption [16]. HMs are categorized based on their function in biological systems as either essential or non-essential HMs. The presence of excessive amounts of essential or non-essential metals can lead to physiological or morphological disorders or genetic mutations [17]. Table 1 categorizes HMs according to their adverse impacts on the human body.

The presence of HMs in the ecosystem is attributed to anthropogenic activities, which are the primary sources of human exposure to these substances [18]. HMs are found in the Earth’s crust in the form of ores, which are extracted as minerals during mining operations. The majority of ores contain HMs, including but not limited to As, Fe, Pb, Zn, gold (Au), Ni, silver (Ag), and Co, in the form of sulfides. Conversely, some ores contain metals such as Mn, Al, and Se in the form of oxides. In the course of mining operations, the extraction process results in the discharge of HMs from the ore, which are subsequently dispersed into the surrounding environment. These metals remain in the soil and may be transported to other locations via air and water currents. Moreover, the utilization of HMs in industrial applications results in the discharge of certain elements into the atmosphere through combustion or into soil and water systems as effluents. Additionally, industrial commodities such as paints, cosmetics, pesticides, and herbicides can also function as origins of heavy metals (Figure 1).

**Figure 2:**
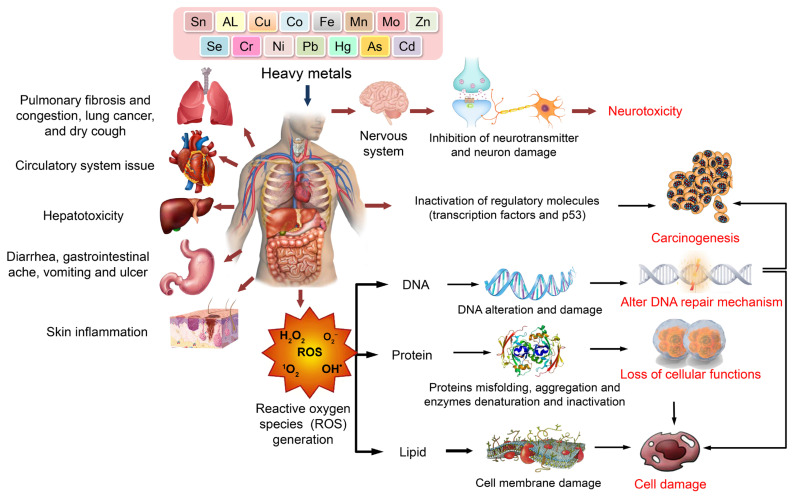
Oxidative stress and human organ toxicity following exposure to HMs.

HMs have the potential to be conveyed to various locations on soil and water bodies via erosion, run-off, or acid rain. The diagram presented in Figure 2 illustrates the trajectory of HM origins and the subsequent impact on human exposure. The toxicity of HMs to biological systems is caused by their ability to form bonds with sulfhydryl groups and generate reactive oxygen species (ROS). The aforementioned phenomenon results in the deactivation of crucial macromolecules, leading to the manifestation of oxidative stress and depletion of glutathione. Upon exposure to toxic metals and subsequent entry into the body, a multitude of processes occur, including the potential interaction or inhibition of certain metabolic pathways [19]. Consequently, a plethora of deleterious impacts on both human and animal populations are observed. The aforementioned conditions encompass a range of medical issues such as organ dysfunction, metabolic irregularities, hormonal imbalances, congenital anomalies, compromised immune system function, and malignancy [20,21]. Consequently, numerous international organizations establish regulations pertaining to the existence of metals in the environment, foods, and drinking water. The subsequent section delineates the origin and toxicity of distinct HMs on human health.

### 2.1. Toxicity in Humans following Exposure to HMs

#### 2.1.1. Effect of Arsenic

Arsenic (As) is a hazardous metallic element that can be found in various geological formations, as well as in water and air. As exhibits carcinogenic properties in humans and is associated with various detrimental health outcomes in both the short and prolonged periods [22]. The process of As biotransformation within the human body involves the methylation of various As compounds, ultimately resulting in the production of detrimental end metabolites such as mono-methylmalonic acid (MMA) and di-methyl arsenic acid (DMA). The thiol group of cells is impacted, leading to impaired functionality in cell enzymes, respiration, and mitotic division [8]. A correlation has been observed between exposure to As and the incidence of cardiovascular disease, including hypertension. The impact of exposure to As and its metabolites, namely MMA and DMA, on hypertension during pregnancy has been reported [23]. The findings revealed that pregnant women with low concentrations of DMA experienced an increase in systolic, diastolic, and main arterial pressure, which could potentially result in adverse cardiovascular health outcomes for both the mother and the child [23].

#### 2.1.2. Effect of Mercury

Mercury (Hg) is a hazardous HM that is ubiquitous in the environment. It has the potential to undergo methylation, leading to the formation of methylmercury (MeHg), which can accumulate in the food web. The consumption of seafood has been linked to human exposure to Hg [24]. Lipoperoxidation and nitrite concentration were found to be elevated while total antioxidant capacity was reduced as a result of the presence of Hg in the forms of HgII and MeHg [25]. Multiple studies have demonstrated that exposure to Hg can have significant adverse effects on children. Specifically, prenatal exposure to Hg has been found to negatively impact child growth, which may be attributed to a decrease in parasympathetic modulation of cardiac autonomic function in children [26]. A positive correlation has been reported between exposure to Hg and blood pressure measures during childhood [27]. There is also a positive association between high levels of Hg and dyslipidemia in adults [28]. Several studies on the effect of Hg on liver function found that liver enzymes increased significantly with Hg exposure [29].

#### 2.1.3. Effect of Lead

Lead (Pb) is a toxic HM that has the tendency to accumulate in various tissues including blood, bones, and most body organs [30]. Exposure to Pb has been associated with various neurological disorders such as Alzheimer’s disease, Parkinson’s disease, amyotrophic lateral sclerosis, and attention-deficit/hyperactivity disorder [31]. There is an observed association between Pd and cardiovascular diseases, and it is regarded as a risk factor for vascular complications in individuals with diabetes [32]. The impact of Pb exposure on liver and kidney functions, as well as on white blood cell count, serum urea, creatinine, aspartate transaminase, alanine transaminase, hemoglobin, and hematocrit concentrations, was observed to be more pronounced in the high blood lead level group as compared to the low blood lead level group [33]. Numerous studies have extensively examined the impact of Pb on children. The findings of these studies have demonstrated that exposure to Pb has adverse effects on the physical growth of children, particularly boys [34]. Exposure to Pd has the potential to alter the concentration of sex hormones, thereby impacting the functioning of the reproductive system [35].

#### 2.1.4. Effect of Chromium

The accumulation of chromium (Cr) in body organs has been found to have potential implications for human health [36]. Cr has a deleterious impact on bronchial epithelial cells, potentially through the aberrant modulation of apoptosis-related proteins, cytoskeletal proteins, and energy metabolism-associated proteins [37]. The impact of Cr on fetal growth during pregnancy has been investigated [38]. The findings of the study suggest that Cr may have a toxic effect on fetal growth. Cr is regarded as a carcinogen that has been linked to the development of lung cancer [39]. The manifestation of hyperpigmented skin is a consequence of exposure to elevated levels of Cr [40].

#### 2.1.5. Effect of Copper

Copper (Cu) is a vital micronutrient for humans. However, excessive levels of Cu can lead to toxic and harmful effects [41]. The accumulation of Cu has been observed to induce mutations and aggregation of mitochondrial protein, leading to a decrease in the activity of the primary antioxidant enzyme and an increase in the production of toxic ROS [42]. A correlation between the elevation of Cu and fibrosis in renal tissues has been established [43]. The presence of Cu has a detrimental impact on male reproductive function, leading to a decrease in sperm count and mobility [44]. There exists a correlation between exposure to copper and obesity [45]. The presence of Cu has an impact on the maintenance of homeostasis of crucial elements such as Ca, Fe, and Mn, which are linked to the induction of oxidative stress. This, in turn, can result in the development of neurodegenerative disorders [46].

#### 2.1.6. Effect of Nickel

Nickel (Ni) is ubiquitously distributed in various environmental compartments such as air, water, and soil [47]. The adverse impacts of Ni on humans have been explored, particularly in the context of pregnancy. The findings revealed a positive association between Ni exposure in pregnant women and preterm delivery [48]. Individuals diagnosed with diabetes exhibited elevated concentrations of Ni in their urine [49]. Offspring may experience congenital heart defects that are associated with maternal exposure to Ni [50]. Ni has been found to have a correlation with immunological disorders, as well as the occurrence of Type I hypersensitivity and Type IV immune reactions in individuals experiencing chronic systemic symptoms associated with Ni [51].

#### 2.1.7. Effect of Uranium

Recent ecological research has examined the potential correlation between prolonged consumption of uranium (U) through drinking water and an elevated risk of leukemia as well as kidney, lung, and colorectal cancer in both genders [52]. It has been evidenced that the absorption of U leads to an increase in ROS, DNA strand breaks, and alterations in gene expression, all of which have adverse clinical consequences [53]. The hexavalent uranyl ion (UO_2_^2+^) has been observed to accumulate in kidney and bone tissues. This accumulation has been found to cause acute and chronic damage to the kidneys, while also increasing the likelihood of osteosarcoma and ontogenesis [54]. Exposure to U either accidentally or through contamination of food or water can lead to a decline in hematopoietic function or bone marrow illness, which can subsequently result in a range of systemic effects [55].

#### 2.1.8. Effect of Cadmium

Cadmium (Cd) is a toxic HM that has detrimental effects on human health [47]. The impact of Cd on the vascular endothelium has been observed to stimulate the release of antithrombolytic substances, leading to the production of various inflammatory indicators [56]. Vijayakumar et al. [57] have conducted a study to ascertain the biological effects of Cd on the proliferation and metastasis of prostate cancer and basal breast tumors. Breast tumor cells may experience a reduction in their anti-oxidative defenses, leading to the initiation of ROS formation upon exposure to Cd [58]. Furthermore, the proximal tubule is the site of Cd accumulation and nephron destruction [59].

#### 2.1.9. Effect of Iron

Iron (Fe) is an essential element for various biological processes, including but not limited to DNA replication, mitochondrial respiration, and oxygen transport, that are crucial for the survival of almost all living organisms. Fe is a redox-active element that plays a role in the generation of ROS, which can cause damage to cellular membranes, DNA, and proteins [60]. Fe is an indispensable constituent of human physiology and participates in numerous cellular metabolic pathways, such as the conveyance of oxygen [61]. Fe deficiency anemia is the most commonly occurring form of anemia worldwide [62]. Fe deficiency can have adverse effects on the development and functioning of the immune system [63]. Fe deficiency during pregnancy can pose risks to both the mother and fetus [64]. In contrast, an overabundance of iron has been associated with heightened susceptibility to cardiovascular disease, gestational diabetes, and fetal complications, as well as the generation of oxidative stress and cellular harm [65].

#### 2.1.10. Effect of Vanadium

Vanadium (V) is present in various environmental compartments such as soil, water, and air. Various tissues and organs, such as the kidney, central nervous system, lung, lymphoid organs, and immune system, undergo histological and physiological changes as a result of this HM [66]. Exposure to V has been associated with various adverse health effects, including respiratory dysfunction, kidney toxicity, biochemical and hematologic changes, toxicity for both reproduction and development, mutagenicity, neurotoxicity, and immunotoxicity [67]. V has an impact on the digestive, respiratory, and cardiovascular systems [68]. The condition known as olfactory dysfunction is the result of exposure to low concentrations of V through the intranasal route. This condition is marked by a decrease in the volume of the olfactory bulb and a decline in dopaminergic neurotransmission to the olfactory bulb [69].

#### 2.1.11. Effect of Cobalt

Cobalt (Co) is a mineral that occurs naturally and can be detected in over a hundred organic and inorganic compounds [70]. The exposure to Co has been associated with inflammations of the upper respiratory tract, including rhinitis and bronchitis, as well as respiratory ailments affecting the lower respiratory system. Simultaneous exposure to certain substances can result in the development of fibrotic alterations in pulmonary tissue, which can subsequently lead to the onset of asthma [71]. Exposure to CO may result in various adverse effects, including lung fibrosis, hepatotoxicity, and carcinogenesis [72]. Exposure to Co can result in various neural system disorders such as memory loss, neuropathies, optic atrophy, and bilateral nerve deafness [73]. The impact of Co on the heart can result in instances of single cardiomyopathy, hypertension, and reversible electrocardiographic alterations [74].

#### 2.1.12. Effect of Thallium

Thallium (TI) exhibits greater toxicity than Hg, Cd, and Pb [75]. Chronic TI poisoning, characterized by symptoms such as anorexia and headaches, can result from prolonged exposure to low levels of TI [76]. Respiratory muscle paralysis may lead to a state of coma in severe instances [77]. One of the characteristic indications of TI poisoning is the occurrence of hair loss subsequent to the contraction of hair follicles. Additional symptoms encompass issues related to digestion, discomfort, mental health, and cardiovascular system complications [78]. The incidence of fetal mortality, preterm birth or low birth weight has been found to be associated with Tl toxicity during pregnancy [79]. 

### 2.2. Mechanism of Intoxication following Exposure to HMs

**Figure 3:**
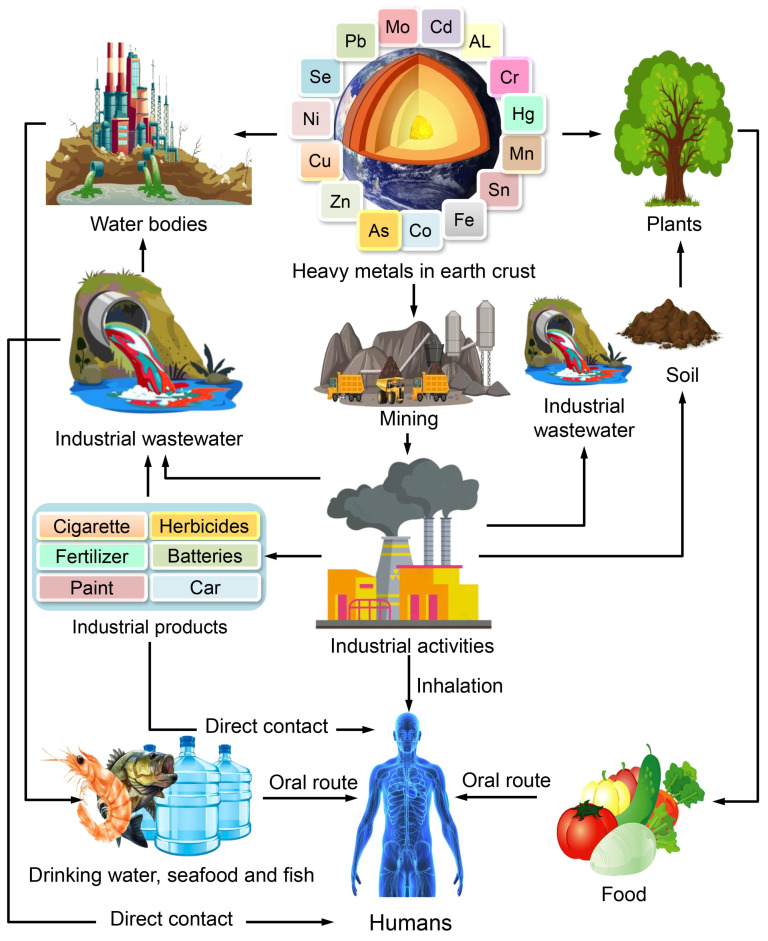
Intoxication mechanisms in humans following exposure to HMs.

Upon ingestion via food or water, HMs undergo acidification within the stomach’s acidic environment. Under acidic conditions, the aforementioned elements undergo oxidation and attain their respective oxidative states (Zn^2+^, As^3+^, Cd^2+^, As^2+^, and Pb^2+^). These states can effectively interact with biological molecules, including proteins and enzymes, to form robust and enduring bonds [80]. The aggregation of proteins may be induced by HM, as evidenced by the observed As-induced protein aggregation, which was found to be dependent on concentration. Furthermore, the aggregates exhibited a diverse array of proteins that were notably enriched in functionalities pertaining to metabolic processes, protein folding, protein synthesis, and stabilization [81]. The ability of these agents to induce protein aggregation in vivo is likely contingent upon their cellular uptake/export efficiency and their unique biological mechanisms. Figure 3 depicts the diverse mechanisms involved in HM intoxication.

## 3. Impact of HMs on Aquatic Animals

**Figure 4:**
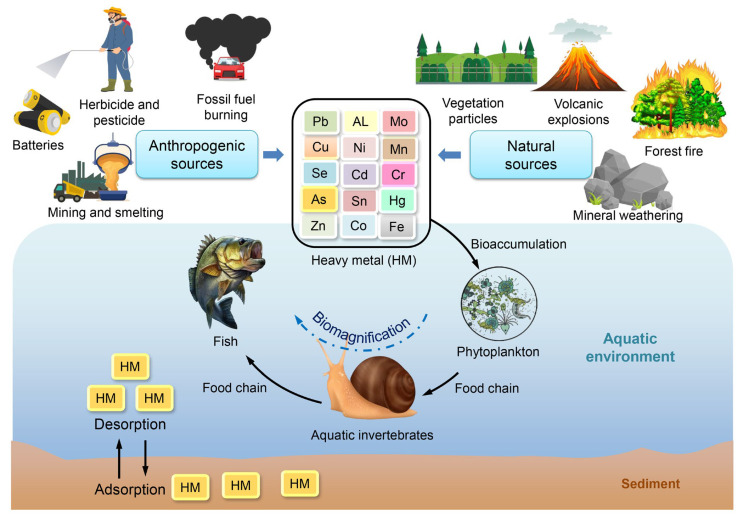
Distribution of HMs in aquatic environments.

HMs are naturally occurring elements that are also generated through human activities, such as industrialization and agriculture. These HMs can enter aquatic ecosystems through direct and indirect means. The transportation of HMs occurs in tandem with the water cycle, whereby they are conveyed from upstream to downstream along river courses, ultimately depositing in vast water bodies such as large lakes, rivers, or oceans [82]. Whilst HMs have the capability to enter water directly, a considerable quantity of HMs are assimilated into the ground and sediment, which function as a conveyance or prospective origin of HMs in aquatic environments [83]. It has been observed that sediment-bound HMs have the potential to migrate into water, and the rate of their release is influenced by various factors such as the type of HMs, as well as the physico-chemical properties of both the water and sediment, including pH, salinity, specific surface area, and cation exchange capacity [83]. Aquatic invertebrates can ingest and accumulate HMs in lakes, rivers, and marine environments through either water or food pathways. HMs have the potential to undergo transfer from aquatic invertebrates to higher trophic levels, ultimately leading to biomagnification. This process can result in harmful consequences for all organisms, including humans. The ingestion of various HMs by aquatic invertebrates has the potential to impact aquatic ecosystems, as depicted in Figure 4.

The biological properties of pure elements and their compounds may exhibit differences in toxicity when considering metals, owing to variations in solubility, oxidation state, and bioavailability. The presence of Cr has been found to impede the growth and reproductive capabilities of aquatic invertebrates, induce oxidative stress and DNA damage, and potentially result in teratogenic and carcinogenic effects [84]. Cu has been found to have various detrimental impacts on aquatic organisms, including but not limited to inducing oxidative stress, modifying enzyme activity, impeding growth or reproduction, interfering with the endocrine system, and diminishing energy acquisition [85]. The exposure of aquatic invertebrates to Zn has the potential to induce oxidative stress and alter various physiological processes, including immune responses, metabolism, and detoxification [86]. As has the potential to cause various physiological and biochemical toxic effects, including but not limited to growth inhibition, oxidative stress, fecundity reduction, apoptosis, and immunotoxicity [87]. Even at low concentrations, Cd demonstrates deleterious impacts on aquatic invertebrates. The impact of Cd resulted in a reduction in the growth rate and reproductive capacity in freshwater fleas. Additionally, the substance caused neuronal toxic effects in the oyster [88]. The presence of Pb in aquatic ecosystems can result in toxicity, which may manifest as the inhibition of crucial enzymatic activity and the occurrence of oxidative stress-induced harm to cell membranes [89]. Hg exhibits a notable attraction towards the sulfur (-SH) group and can readily attach to the cysteine component found in proteins. This phenomenon leads to functional impairment and toxicity in aquatic invertebrates [90].

## 4. Impact of HMs on Soil and Plants

**Figure 5:**
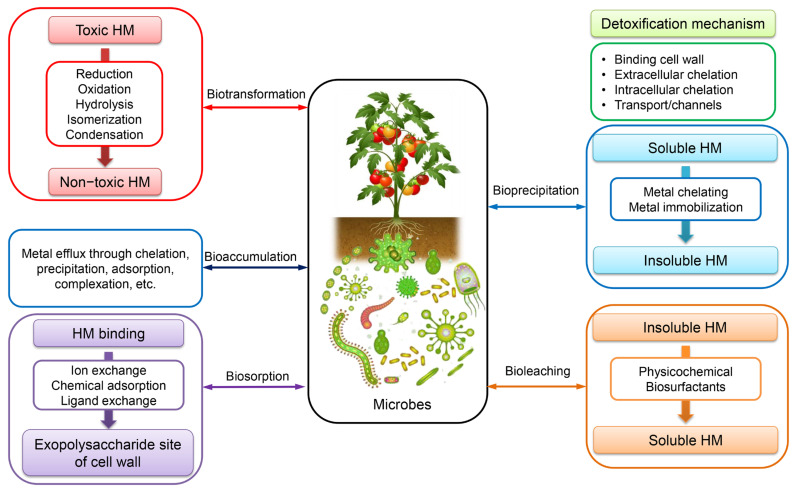
Microbial remediation mechanisms of soils contaminated with HMs.

The stress induced by HMs is a significant factor that adversely impacts the agricultural yield of plants. HMs are widely acknowledged as a constituent of soil. However, their concentration in soil and plants can have detrimental effects on the environment [91]. HMs have the ability to transfer through the food chain after being absorbed by plants, ultimately accumulating in the bodies of animals and humans and posing potential health risks [92]. HMs have the potential to infiltrate groundwater or surface waters and subsequently undergo absorption by plants or turn into emissions as gases that are sent into the atmosphere. Alternatively, they may form semi-permanent associations with soil constituents such as clay or organic matter, ultimately impacting human health at a later time. It is imperative to keep soil pollution levels below the prescribed toxicity thresholds to ensure sustainability [93]. The utilization of chemical fertilizers and pesticides has been found to elevate the likelihood of soil contamination by HMs, which can subsequently accumulate in the tissue of crops cultivated in such contaminated soil [94]. The mobility of HMs in soil is significantly influenced by the presence of total organic matter and pH levels. Upon introduction of HMs to soil, their downward movement is limited by the soil’s capacity to retain the metals or by the interaction of the metals with the adjacent waste matrix, which may enhance their mobility. High concentrations of HMs in soil can have adverse effects on the development, growth, and nitrogen fixation of legumes [95]. Microbial remediation refers to the biological process by which microorganisms adsorb or transform heavy metals into less toxic products. Microbial remediation is a process that primarily involves various mechanisms such as biosorption, bioaccumulation, bioleaching, biovolatilization, and biomineralization [96], as illustrated in Figure 5, for the purpose of addressing HM contamination in soil.

There was a decrease in the concentration of HMs in the soil as the depth increased [97]. The transport of HMs ions is influenced by both their properties and the texture of the soil. The duration required for the infiltration of HMs in sandy and compound soil is comparatively shorter than that in loess. The migration resistance of loess to all metals is significantly higher when compared to sandy and compound soil. HMs are immobilized in the soil through the formation of complexes with ligands, which can be either organic or inorganic in nature. When subjected to leaching conditions, HMs present in soil have the potential to contaminate surface water and groundwater, thereby exacerbating water pollution and adversely impacting aquatic organisms [98].

The detection of Hg in soil solutions poses a challenge owing to its robust affinity with soil particles and organic substances. Inorganic Hg can cause toxicity to humans and disrupt soil biota across all trophic levels [99]. The conversion of Hg^2+^ in soil into methylmercury, a highly toxic substance that accumulates in plants and is subsequently enriched in the food chain, poses a significant risk to human health through chronic poisoning. This process occurs through various mechanisms, including absorption, migration, and transformation [100]. The presence of Hg in soil has a notable effect on both the nitrogen cycle of the soil and the prevalence of microorganisms inhabiting the soil [99].

Elevated concentrations of Cu have deleterious effects on various metabolic pathways in plants, including but not limited to photosynthesis, respiration, and enzymatic activity. Cu has been found to enhance the production of ROS in plants and upregulate the expression of genes associated with oxidative stress response [101]. Elevated concentrations of Cd have a significant impact on the development of soil microorganisms and the activities of enzymes. In contrast to the majority of other metals, it has been observed that Cd exhibits a higher degree of plant absorption and can translocate into the edible portions prior to the onset of phytotoxicity [91].

Cr is a noxious element for plants. Cr is a naturally occurring element that is ubiquitously distributed in the environment, encompassing the soil, air, and water compartments [102]. Cr is present in two oxidation states, namely trivalent and hexavalent, in soil. However, hexavalent Cr is comparatively more toxic and mobile than its trivalent counterpart [103]. The presence of Cr in soil has a negative impact on both soil fertility and microbial activity, ultimately resulting in decreased plant yield [104]. The presence of Cr compounds can have detrimental effects on the growth and development of plants. This can manifest in altered germination processes and hindered development of roots, stems, and leaves, ultimately leading to reduced yield and dry matter production [105]. The elevated concentration of soil Pb has been associated with certain chemical fertilizer production enterprises. Pb has a tendency to accumulate in the roots and lower above-ground portions of plants. While plant roots are not highly receptive to Pb absorption, the presence of this element can negatively impact various metabolic processes in plants, including root development, photosynthesis, and water uptake [93]. The enzymatic activity of soil biota is hindered by Pb, leading to the buildup of partially digested organic matter [106].

**Figure 6:**
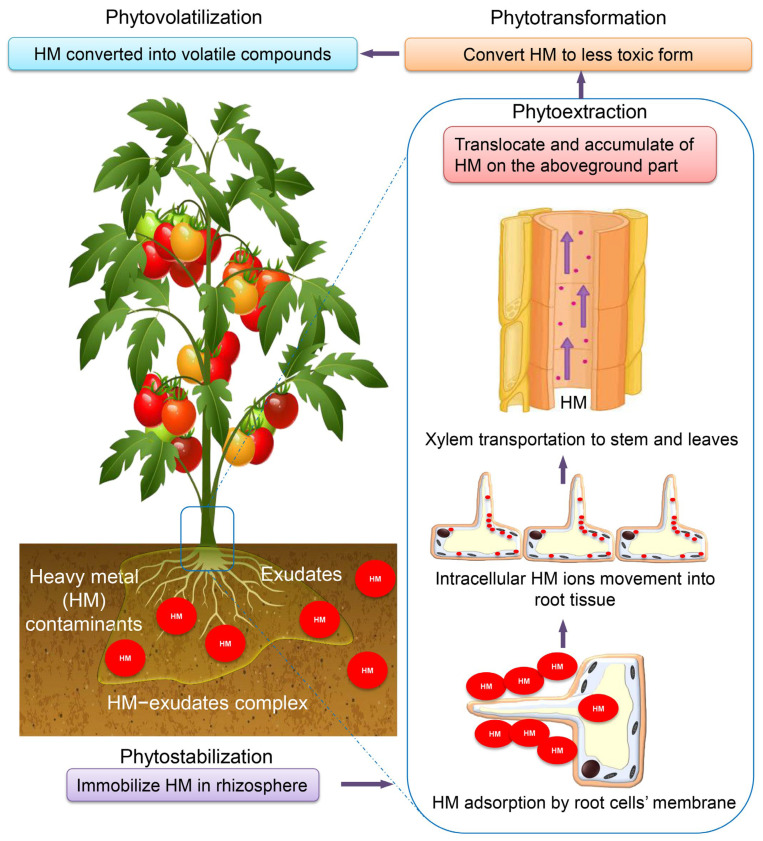
Transfer route and phytoremediation mechanism of HMs into plant cells and tissues.

Phytoremediation techniques represent an environmentally friendly technology that facilitates the detoxification of polluted metal-lands, water, and groundwater through the implementation of various processes such as phytoextraction, phytostabilization, phytovolatilization, rhizofiltration, and phytofiltration [107]. The utilization of plants for the remediation of contaminated sites, known as phytoremediation, is a sustainable approach that has been extensively investigated in both field and laboratory settings. HMs exhibit resistance to remediation through both microbial and physiological mechanisms, and they persist in soil for extended periods, thereby posing a potential threat to the ecosystem [108]. It is imperative to implement remedial measures to mitigate the consumption of metals by the atmospheric, terrestrial, and aquatic ecosystems, and to curtail the contamination of groundwater [109]. Numerous phytoremediation techniques entail the utilization of rhizodegradation and phytodegradation mechanisms to facilitate the decomposition of both organic and inorganic pollutants. The diagram presented in Figure 6 illustrates the implementation of phytoremediation as a means of addressing the issue of HMs in plants.

## 5. Toxic Effect of HMs on Microbial Cells

**Figure 7:**
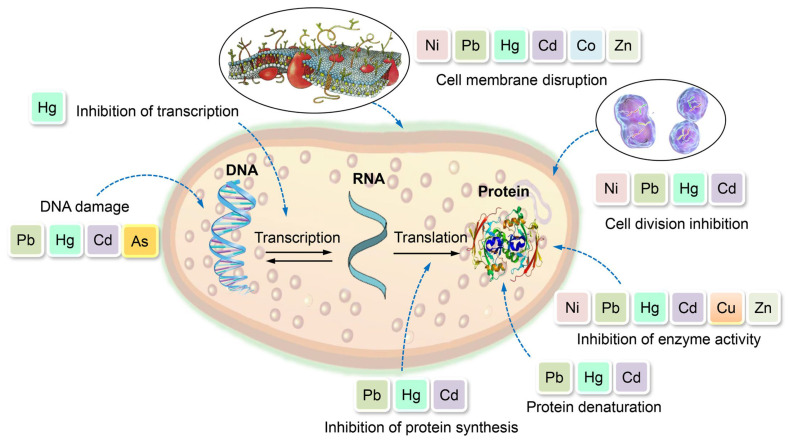
Toxicity of HMs on microorganisms.

The toxicity of HMs is attributed to multiple mechanisms, which include degradation of enzymatic functions leading to lethality, functioning as redox catalysts in the generation of ROS, disruption of ion regulation, and direct effects on the composition of DNA and proteins. Cu has the ability to catalyze the production of ROS, thereby serving as soluble electron carriers. The aforementioned phenomenon has the potential to induce significant harm to cytoplasmic molecules, DNA, lipids, and other proteins [110]. HMs have an impact on the rate of enzyme activity, which is influenced by various soil factors such as pH, organic content, and chemical composition. The negative effects of toxic metal-contaminated soil can be attributed to the decrease in urease, catalase, and lipolytic activity. There exists an inverse correlation between the concentration of HMs and soil enzyme activity. A decrease in bioavailability of Cd, Cu, and Pb in treated soil results in an increase in soil enzyme activity [111]. Figure 7 illustrates the adverse impacts of HMs on microbial cells. Cr has the potential to induce oxidative harm and denaturation of microorganisms, thereby impeding their bioremediation efficacy. The electric interaction between the negatively charged phosphate groups of DNA and intracellular cationic Cr complexes could potentially affect transcription and replication processes, leading to mutagenesis [110]. Both Cd and Pb exhibit deleterious impacts on microorganisms, causing harm to their cell membranes and disrupting the integrity of their DNA structure. The aforementioned impairment arises due to the dislodgment of metallic elements from their initial binding locations or bonding interactions [112]. The manipulation of DNA structure can have an impact on microbial morphology, metabolism, and growth by inducing functional disruption, cell membrane disruption, inhibition of enzyme activity, and oxidative phosphorylation [113].

## 6. Methods for the Remediation of HMs

Removal of HMs is typically accomplished by physical remediation, chemical remediation, and bioremediation [114,115,116,117,118,119]. The advantages and disadvantages of remediation technologies are summarized in Table 2.

## 7. Bioremediation: Interaction of Microorganisms with HMs

**Figure 8:**
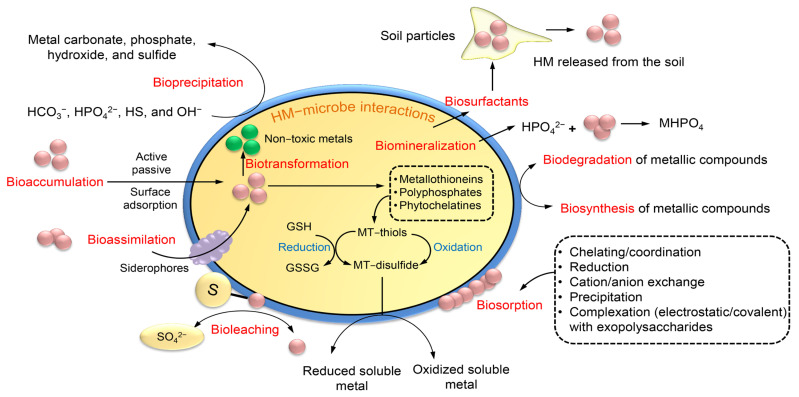
Mechanisms of microbial bioremediation of heavy metals. GSH, glutathione; GSSG, glutathione disulfide; MT, Metallothionein.

Bioremediation is an innovative technology that employs a variety of biological agents, including bacteria, fungi, algae, yeasts, moulds, and plants, to effectively eliminate, detoxify, transform, or neutralise the adverse effects of HMs [120]. In contrast to several physicochemical techniques commonly employed for mitigating HM pollution, bioremediation presents a variety of economically viable advantages owing to its notable efficacy in HM removal, cost-efficiency, ease of management, and widespread availability in both contaminated soil and water [121]. Microorganisms are a significant factor in the bioremediation of HMs among biological agents. These organisms are not only proficient in the dissolution of HMs, but also actively participate in the oxidation and reduction of transition HMs. The utilisation of microorganisms’ metabolic abilities for the eradication of HM pollution is a form of green technology. Overall, most HMs have been classified as toxic. However, various biological organisms have developed distinct resistance mechanisms and intricate intracellular pathways to facilitate the utilization, interaction, adaptation, and detoxification of HMs for the purpose of cellular regeneration [122]. Figure 8 illustrates the various mechanisms employed by microorganisms for the elimination of HMs, including detoxification, biosorption, degradation, mineralization, and transformation from highly toxic to low toxic forms [122,123]. Furthermore, it has been suggested that the utilization of fungi and bacteria may have a potential role in the bioremediation of HM, as depicted in Table 3 [124,125,126,127,128,129,130,131,132,133,134,135,136,137,138,139,140].

### 7.1. Mechanisms of HM Removal by Fungi

**Figure 9:**
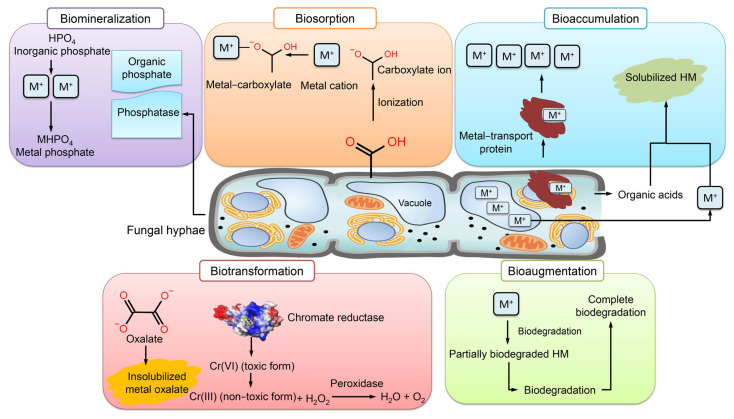
Mechanisms of HM removal by fungi.

The most common mechanisms of HM mycoremediation are bioaugmentation, bioaccumulation, biosorption, biomineralization, and biotransformation [41] (Figure 9). The implementation of fungi in bioaugmentation exhibits the most superior performance in terms of both metal elimination and enzymatic activity [141]. Bioaccumulation is a biological phenomenon wherein microorganisms synthesize proteins to recycle HMs for utilization in various cellular processes such as enzyme catalysis, signaling, and stabilization of charges in biomolecules [142]. Fungi have the ability to mitigate the harmful impact of HMs by generating inorganic acids and organic acid complexes that facilitate the leaching of HMs. This characteristic makes fungi a promising candidate for the sustainable management of wastewater. Certain types of fungi have the ability to synthesize metal transport proteins that facilitate the efflux of heavy metals from the cell or their sequestration into vacuoles [143]. The elevated levels of proteins resulting from the heightened concentration of iron facilitate the synthesis of crucial proteins that participate in the detoxification processes of *Aspergillus flavus*, thereby mitigating the deleterious effects of iron [144].

Biosorption has emerged as a viable alternative for HM remediation. Fungi are known to primarily employ this technique through the ion exchange mechanism that occurs between their sorption materials or various polymeric substances, such as EPS [145]. The present approach involves the utilization of adsorbents as biological agents that facilitate the binding of HMs to non-living biomass of microorganisms, particularly through the functional groups of cell wall sorption components. The binding of metal ions is contingent upon the pH level, which influences the electrostatic interaction between metal ions and a functional group. At low pH levels, two distinct phenomena take place: firstly, the functional groups carried by the mycelium undergo ionization, resulting in the generation of a negative charge. Secondly, the occurrence of protonation in a solution can be attributed to the presence of free hydrogen ions resulting from an acidic pH. As a result of these two mechanisms, a significant quantity of unbound protons present in the solution adhere to the negatively charged sites on the cellular wall of the fungus [145]. Under optimal pH conditions, the fungus exhibits an elevated binding affinity and establishes electrostatic interactions with positively charged metal ions. The influx of metal complexes into the fungal cell results in a decrease in the quantity of metal ions absorbed from the solution, thereby affecting the fungus’s retention capacity and ultimately leading to a reduction in its biosorption potential [146]. The augmentation of the incubation period results in an elevation of both biomass production and functional groups, leading to a corresponding increase in the quantity of biosorbent metal attached to said groups.

The metal that is biosorbed in the fungal cell wall is bound by metal-chelating proteins, and subsequently either restored in vacuoles or precipitated in the fungal inner cell surface through the formation of intracellular organic acid [41]. The process of metal binding to cell surface binding sites is contingent upon a variety of factors, including the intricate structure of microorganisms, extracellular precipitation, intracellular accumulation, oxidation and reduction, and methylation/demethylation [147]. Fungi possess inner walls with multiple layers on their adsorbent surface, which are accountable for the sorption of metals. As a result of metal adsorption, the surface of fungi appears rough. The fungal surface is comprised of a capsule layer that is characterized by the presence of carboxyl groups. These groups are instrumental in facilitating the mechanism of metal biosorption [148]. Metal cations can effectively bind to the fungal surface with the aid of anionic ligands, including carboxyl and amino groups. Metal ions exhibit an affinity for functional groups containing atoms with electron-donating properties. The carboxyl group of galacturonic acid, which is the primary constituent of fungal capsules, facilitates cation binding by attracting charges. The presence of oxygen in carboxylate ions facilitates anionic binding and enhances the attraction toward metal cations [148].

The process of biomineralization pertains to the capacity of living organisms to form minerals as a means of mitigating the harmful effects of metal toxicity. The biomineralization of metal phosphate is initiated by the production of acid phosphatase by fungi in a medium containing phosphate, leading to the formation of phosphate binders [149]. Certain fungi exhibit uneven diffusion of metals such as Pb^+2^ in their mycelia, resulting in the manifestation of green fluorescence with granular distribution. This phenomenon is attributed to the biomineralization of metals [150]. The concentration of precipitated calcium carbonate increases concomitantly with the rise in calcium oxide levels within the fungal culture inhabiting the contaminated concrete cube. The aforementioned process induces ion activity, thereby facilitating the mineralization of the solution, leading to the substantial precipitation of calcium carbonate. The entrapment of Cr by *Candida orthopsilosis* resulting in the formation of calcium carbonate precipitate has been observed to enhance the removal of HM and facilitate the mineralization of the surfaces of concrete cubes [151].

Biotransformation involves the conversion of toxic metal forms into less toxic forms through oxidation–reduction or mineralization–demineralization reactions [152]. The capacity of fungi to generate oxalate, which combines with HMs to form coordination compounds that function as chelators, enables them to convert soluble metals into insoluble particles, specifically insoluble metal oxalates [145]. A range of fungi have the ability to undergo biotransformation of chromium, arsenic, and other HMs into non-toxic forms. Additionally, these fungi are capable of accumulating the transformed metals in their hyphae, thereby preventing the entry of toxic metals into the roots of plants [153].

### 7.2. Mechanisms of HM Removal by Bacteria

**Figure 10:**
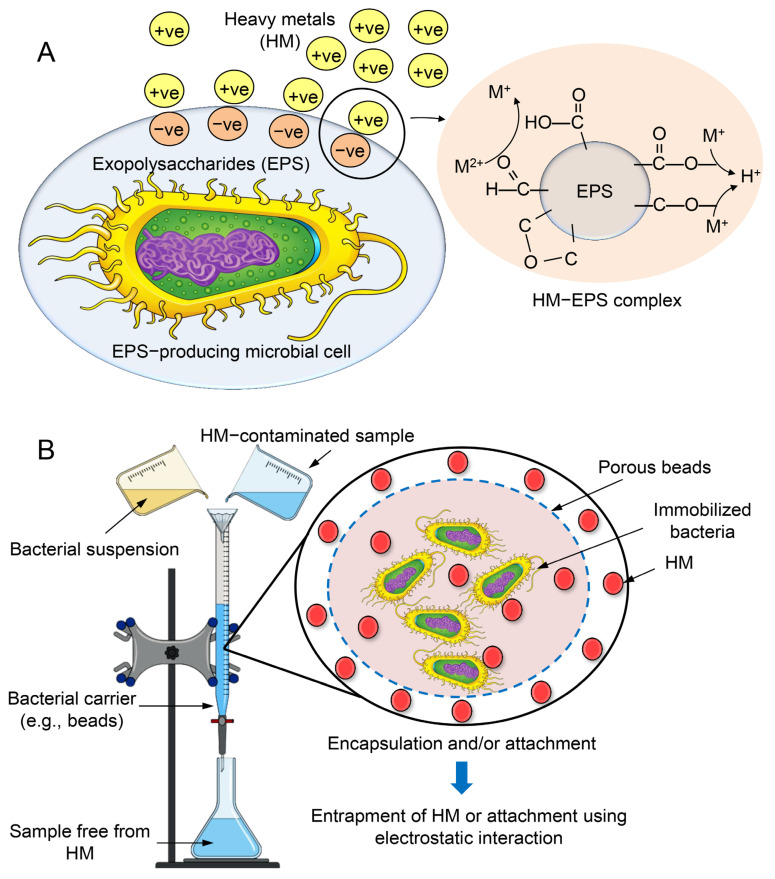
Mechanisms of bacterial exopolysaccharide (**A**) and encapsulation (**B**) for HM removal.

Biosorption is a biological physicochemical process that is employed for the removal of recalcitrant compounds, including metal ions [112]. The utilization of this approach is characterized by its cost-effectiveness, high efficiency in eliminating toxicity, absence of the requirement for supplementary nutrients, and minimal production of chemical and/or biological sludge [112]. The analysis of the supernatant derived from suspension cultures of bacteria revealed that the primary factor responsible for metal sequestration was the soluble exopolysaccharides (EPSs). EPS is composed of carbohydrates and proteins, which jointly facilitate the biosorption of Zn and Pb [154]. The secretion of EPS has been observed in various bacterial strains, including *Paenibacillus jamilae*, *Bacillus firmus*, *Bacillus licheniformis* KX657843, *Herbaspirillium* sp., and *Paenibacillus peoriae* TS7. The biosorption capacity of the substance in question exhibited notable efficacy in the elimination of various metals, including Zn^2+^, Pb^2+^, Ni^2+^, Cu^2+^, Cd^2+^, Co^2+^, and Hg. This can be attributed to the electrostatic interactions that occur between the negatively charged functional groups of EPS and the positively charged metal ion (Figure 10A) [155]. The biosorption process is primarily influenced by the quantity of biosorbent utilized. Bacterial immobilization is a biological process that relies on the utilization of bacteria’s inherent characteristics to eliminate various pollutants. The fundamental principle of the mechanism involves the interplay between bacteria and HMs, facilitated by the immobilization of the biocatalyst onto diverse materials that serve as carriers, providing safeguarding against external factors. This immobilization enables the microbial cells to be readily available for degradation purposes [156]. Two mechanisms, namely the attachment method and the encapsulation method, can be utilized to elucidate bacterial immobilization mechanisms, as depicted in Figure 10B.

**Figure 11:**
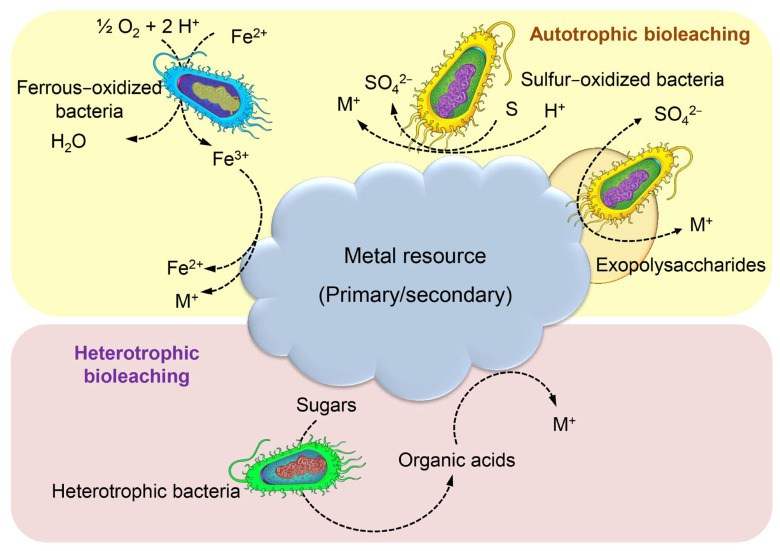
Bacterial bioleaching mechanism for HM removal.

Bioleaching is a microbial-mediated process employed for the purpose of solubilizing minerals from waste solids. The process of bioleaching has been found to be both cost-effective and efficient in the removal of a diverse range of HMs, including but not limited to Cd, Cu, Cr, Fe, Ni, Pb, and Zn. The involvement of mesophilic bacteria in metal leaching is attributed to their ability to generate acids such as sulphuric acid, which facilitate the mobilization of metals. The reduction in pH and increase in oxidation–reduction potential (ORP) can effectively create an optimal environment for the removal of heavy metals (HMs) from sludge [157]. Sulfur-oxidizing and iron-oxidizing bacteria are the predominant bacterial strains employed in bioleaching [8]. The process of bioleaching can occur either through the direct metabolic activity of leaching bacteria or indirectly through the by-products of bacterial metabolism [8]. The direct mechanism involves the binding of bacteria with HMs, leading to the oxidation of the latter to their soluble metal forms. Bacteria have been observed to convert sulphur to sulfuric acid through an indirect mechanism. This process results in a reduction in the pH of the medium and an increase in the solubility of HMs, as demonstrated in Figure 11 [157]. Biomineralization refers to the process by which various solid minerals, such as carbonates, phosphates, silicates, and sulphates are formed from metal ions that are subsequently precipitated by microorganisms that are actively involved in the process. Microbial-induced precipitation is the governing mechanism of biomineralization, which encompasses microbial-induced carbonate perception that is contingent upon urea hydrolysis, pH, and temperature [135]. The process of biomineralization involves the participation of various microorganisms, including photosynthetic microorganisms and sulfate-reducing bacteria, which contribute significantly to mineral precipitation through autotrophic and heterotrophic pathways [158].

### 7.3. Advances in Microbial Technologies for HM Removal

Extensive endeavors have been undertaken over a prolonged period to tackle the protracted persistence of HMs in the environment. Notwithstanding, the issue persists as a result of the increasing utilization of HM and the absence of feasible eradication remedies [159]. In order to mitigate the potential ecological ramifications of HM pollution, it is imperative to develop innovative and robust ecological technologies that can effectively extract and reclaim HMs from contaminated environments. The utilization of microbial bioremediation has been identified as a cost-effective approach to address the issue of HM contamination in environmental sites. Several techniques that hold promise for the removal of HMs from soil, including microbial fuel cells, biofilm, nanotechnology, and genetic engineering have been identified [160].

#### 7.3.1. Microbial Fuel Cells

**Figure 12:**
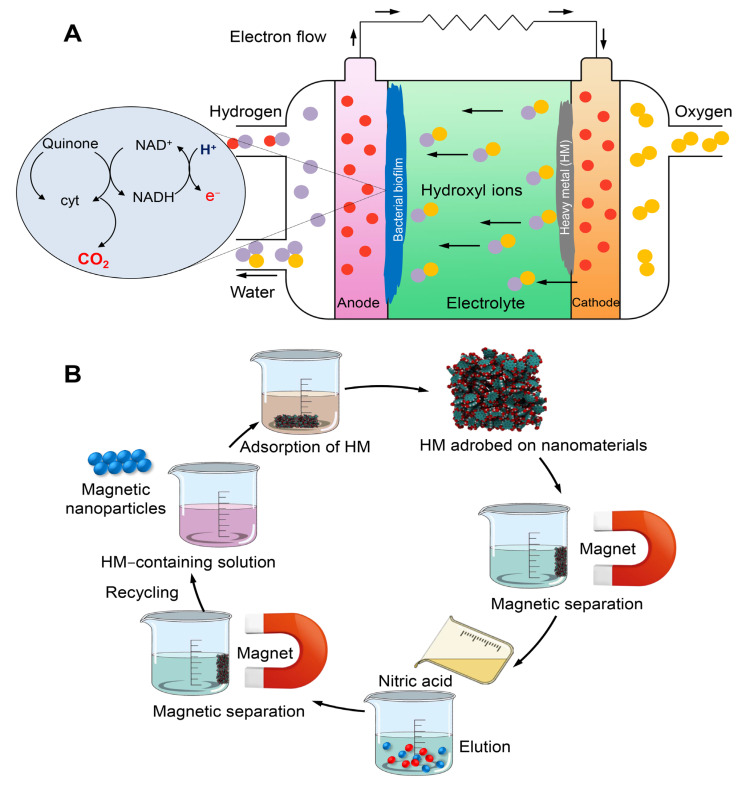
Mechanisms of HM removal via microbial fuel cells (**A**) and adsorption of metal ions across mesoporous magnetite nanoparticles (**B**).

Microbial fuel cells (MFCs) are bio-electrochemical devices that facilitate the decomposition of organic waste into smaller molecules, thereby liberating protons (H^+^) and electrons (e^−^), which subsequently culminates in the production of energy. The diagram presented in Figure 12A illustrates the general structure and functioning of the MFC. Microorganisms generate electrons and protons at the anode through the process of oxidizing organic materials. Additionally, these microorganisms assimilate metal ions into their biomass. The transportation of electrons occurs through an external circuit, while the diffusion of protons toward the cathode takes place through the solution [161]. In principle, it is possible for any chemical possessing a high redox potential to function as the cathodic electron acceptor within the context of an MFC. Certain heme molecules exhibit significantly elevated redox potentials. Hence, they can serve as electron acceptors, wherein metallic ions amalgamate with protons and undergo reduction, thereby facilitating their elimination from the HMs and subsequent retrieval [162].

MFCs have been identified as a feasible approach to mitigate environmental contamination. The proposed approach presents a viable remedy for the treatment of wastewater and soil, along with the removal and recovery of HMs [163]. MFCs have the capability to convert chemical energy into electrical energy via oxidation mechanisms facilitated by microorganisms or enzymatic catalysis. MFCs provide a number of advantages in comparison to conventional fuel cells. The production of fuel can be achieved through the utilization of diverse organic or inorganic materials, including but not limited to soil sediments and organic waste [164]. The utilization of this technology has the potential to offer significant benefits in regions lacking access to electrical power and to improve soil characteristics. Notwithstanding its potential benefits, the technology presents a number of obstacles, including suboptimal rates of electrical generation. Consequently, various research endeavors have been undertaken to enhance electrical efficiency [165]. The removal of HMs from wastewater and soil has been the subject of extensive research utilizing MFC-based techniques [164,165,166]. Given the limited power output of MFCs, it is advisable to employ them primarily as sensors for detecting the toxicity of HMs [167].

#### 7.3.2. Biofilm Technology

The microbial communities comprising bacteria, fungi, microalgae, and yeast are crucial in mitigating the deleterious impact of inorganic metallic salts, HMs, and organic waste on soil and water quality [168]. The replacement of physicochemical procedures with microbial metabolism for efficient utilization is a more environmentally friendly, secure, and effective strategy [169]. The microbial population generates EPSs, which enhance the efficacy of HM elimination. EPS substances are a class of biogenic macromolecules composed of proteins, uronic acid, and polysaccharides. These compounds are synthesized by microorganisms as a mechanism of self-preservation in response to various environmental stressors, such as extreme temperatures, acidic or alkaline conditions, and other physiological challenges. The aforementioned stressors enhance the efficacy of metal sequestration in the bioremediation of heavy metals [169]. The formation of biofilms is a consequence of the association of one or more species, and the composition and configuration of EPS can be influenced by environmental factors [170]. EPS contains a significant amount of anionic charge. The sequestration of metallic ions occurs in substantial quantities [171]. The utilization of microbial biofilm has demonstrated a high degree of efficacy in the removal of HMs from both wastewater and soil.

#### 7.3.3. Nanomaterial for HM Removal

Carbon nanotubes (CNTs) have demonstrated exceptional adsorption capabilities toward various HMs such as Cu, Pb, and Cr, owing to their potential adsorption active sites. The utilization of CNTs for the purpose of removing HMs from wastewater is impeded by the considerable expenses associated with their production, as well as the difficulty of isolating CNTs from the wastewater stream [172]. The utilization of silica-based nanomaterials has been investigated as a means of extracting HMs from polluted sites, owing to their exceptional surface properties [173]. The promotion of HM adsorption can be achieved through the utilization of nano-silica as the foundation for nanocomposites or by modifying its surface with functional groups such as -NH2 and -SH. Silica-based nanomaterials possess remarkable surface properties and are non-toxic, which makes them advantageous [174]. The employment of nanotechnology exhibits considerable potential in the realm of environmental remediation, particularly in the removal of HMs. The manipulation of material properties has been utilized for the purpose of removing HMs from the environment. The material exhibits exceptional surface properties and quantum effects due to its reduction to the nanoscale [172]. The principal modality by which nanomaterials eliminate HMs is through their elevated adsorption capacity and discernment for HMs (Figure 12B).

#### 7.3.4. Biotechnological Tools

The application of contemporary biotechnological techniques such as genetic engineering and recombinant DNA technology has expedited the process of biodegradation and remediation of HMs [173]. Metagenomics and metabolic investigations offer insights into microbial diversity, population, and functional composition with respect to metal resistance genes. These findings can be leveraged to improve the efficacy of microbial strains in the removal or degradation of heavy metals. The field of genetic engineering has enabled the transfer of advantageous traits from one species to another, resulting in the development of specific strains for the purpose of bioremediating soil, sludge, or polluted water [175].

## 8. Conclusions

The issue of toxicity caused by HMs has emerged as a significant environmental challenge, with detrimental impacts on agricultural productivity and food security. The toxic effects of HMs result in their accumulation in the environment, which has adverse effects on human health, plant growth, and marine ecosystems. HMs can gain entry into the human body through various routes, such as ingestion via drinking water or food, inhalation through air, or through dermal exposure. After being absorbed, HMs are retained and accumulate within the human body. The accumulation of noxious metals in biological systems results in a range of deleterious consequences on diverse body tissues and organs. Various physical and chemical approaches have been suggested as potential remedies for the pollution caused by HMs. Nonetheless, the effectiveness of these techniques has been limited, and progress in this area has been slow. Therefore, alternative methodologies utilizing bioremediation have been explored. The employment of microbial bioremediation has emerged as an effective, reliable, and eco-friendly alternative for HM detoxification. However, HMs can interfere with the cellular activities of microorganisms depending on the existing concentration. The metal-tolerant microbes can enhance HM bioremediation. Microbes exhibit varying cellular structures, and their mechanisms for dealing with HM toxicity are typically dependent on the affinity of cellular biomolecules for metal ions and the stability of the specific metal in question. Microorganisms employ enzymatic systems or develop resistance through various mechanisms as a means of self-protection against HMs. However, the development of large-scale applications of combined remediation technology is recommended. In the context of practical application, one can acquire knowledge from both domestic and international experiences and enhance the judicious utilization of cutting-edge technologies. The mitigation of harmful effects caused by HMs necessitates the implementation of both scientific and community-based remediation technologies. Furthermore, forthcoming studies will derive advantages from assessing novel targets as protective procedures against organ toxicity triggered by HMs.

## Figures and Tables

**Table 1:** Classification of HMs based on their impact on the human body.

HM Compounds	Impact HM on Human
Lead (Pb), mercury (Hg), arsenic (As), and cadmium (Cd)	High degree of toxicity, carcinogenic, and induce damage to multiple organs
Tin (Sn) and aluminum (Al)	Less toxic to human body
Barium (Ba) and lithium (Li)	Non-essential for physiological and biochemical functions
Copper (Cu), zinc (Zn), nickel (Ni), manganese (Mn), molybdenum (Mo), cobalt (Co), selenium (Se), chromium (Cr), and iron (Fe)	Essential for physiological and biochemical functions

**Table 2:** Remediation approaches of HMs.

Remediation Type	Approach	HM	Advantages	Disadvantages	References
Physical	Replacement	Ni, Cd, Cr, Zn, Cu, Pb	Appropriate for smaller contaminated sites	Expensive	[114]
	Thermal desorption	Hg	Effective Safe Lack of secondary pollutants Short pretreatment period	Expensive devices Long desorption time	[115]
Chemical	Oxidation/reduction	Cu, As, Sb, Cr, Pb, Se	Suitable for soils that are heavily polluted with HMs	Expensive Need large quantity of chemicals	[116]
	Leaching	Cr, Zn, Cu, Pb, Ni, Cd	Suitable for soils that are heavily polluted with HMs	Formation of secondary pollutants	[117]
	Electrokinetic remediation-permeable reactive barrier	Cd, Pb, Cr, Ni	Suitable for soils that are heavily polluted with HMs	Time-consuming	[118]
	Stabilization	Pb, Cd, Cu, Mn, Zn, As, Fe, Ni	Effective Low cost Convenient	Pollutants cannot be removed thoroughly	[118]
Bioremediation	Microbial remediation	Cr, Pb, Ni, Zn, As,	Effective Low cost Lack of secondary pollutants	Concentration limit Time-consuming	[119]
	Phytoremediation	Se, Cd, Cu, Pb, Zn	Convenient Eco-friendly Low cost	Time-consuming Non-specific Low efficiency	[119]

**Table 3:** Efficient bacterial and fungal species used in HM removal.

Microorganisms	HM	Efficiency of Removal (%)	References
*Penicillium* spp. XK10	Cd	32.2	[124]
*Cladosporium* sp. XM01	Mn	91.5	[125]
*Penicillium janthinillum* GXCR	Pb, Fe, Cu	85–99	[126]
*Mucor rouxii*	Pb, Zn,	11–35	[127]
*Aspergilus niger*	Cd, Cr	43–98	[128]
*Aspergillus flavus*	As, Pb, Cr, Ni	46–97	[129]
*Trichoderma brevicompactum*	Cu, Cr, Cd, Zn	4–64	[130]
*Aspergillus fumigatus*	Cd, Cr	69–79	[131]
*Penicillium simplicissimum*	Cr, Pb, Cu, Cd, Zn	28–88	[131]
*Trichoderma virens*	Cu, Cr	63–70	[132]
*Pseudomonas putida*	Cu	25.4	[133]
*Bacillus subtilis*	Cu	37.8	[133]
*Pseudomonas aeruginosa*	Hg	60	[134]
*Sporosarcina pastaurii*	Cd, Zn, Pb	94–98	[135]
*Variovorax boronicumulans*	Cd, Zn, Pb	73–95	[135]
*Stenotrophomonas rhizophila*	Cd, Zn, Pb	63–96	[135]
*Citrobacter freundii*	Cd, Cu, Fe, Mn, Zn	40–80	[136]
*Pseudomonas aeruginosa*	Cu, Zn	18–65	[137]
*Acinetobacter* sp. B9	Ni	69	[138]
*Bacillus thuringiensis*	Hg	62	[139]
*Streptomyces* sp.	Pb	83	[140]

## Data Availability

Data will be given upon request.
